# Gene expression pattern of TCR repertoire and alteration expression of IL-17A gene of γδ T cells in patients with acute myocardial infarction

**DOI:** 10.1186/s12967-018-1567-7

**Published:** 2018-07-05

**Authors:** Xiao-ming Chen, Tao Zhang, Dan Qiu, Jian-yi Feng, Zhen-yi Jin, Qiang Luo, Xin-yu Wang, Xiu-li Wu

**Affiliations:** 1 Department of Cardiology, The First Affiliated Hospital of Jinan University, Guangzhou, 510630 China; 2 Institute of Hematology, Jinan University, Guangzhou, 510632 China; 3 Peking University Institute of Hematology, Peking University People’s Hospital, Beijing, 100044 China

**Keywords:** γδ T cells, Acute myocardial infarction, IL-17A, Foxp3

## Abstract

**Background:**

γδ T cells are associated with the pathogenesis of coronary atherosclerotic heart disease, but the relationship between the development of acute myocardial infarction (AMI) and γδ T cells is not clear. So we attempt to investigate the expression pattern and clonality of T cell receptor (TCR) repertoire of γδ T cells in AMI patients, analyze the expression levels of regulatory genes Foxp3 and IL-17A, and characterize the correlation between γδ T cells and the pathogenesis of AMI.

**Methods:**

25 patients diagnosed with ST-segment-elevation AMI were enrolled and 14 healthy individuals were recruited as the controls. RT-PCR and GeneScan were used to analyze the complementarity-determining region 3 sizes of TCR γδ repertoire genes in sorted γδ T cells from peripheral blood mononuclear cells (PBMCs). RQ-PCR was used to detect the gene expression levels of Foxp3, IL-17A and TCR Vγ subfamilies in sorted γδ T cells. All the patients were followed up for recordings of clinical endpoints.

**Results:**

The mRNA gene expression levels of TCR Vγ1, Vγ2, and Vγ3 subfamilies in AMI patients were significantly higher than those in healthy controls. The expression pattern was Vγ1 > Vγ2 > Vγ3 in AMI patients, while Vγ1 > Vγ3 > Vγ2 in healthy controls. The significantly restricted expression of TCR Vδ subfamilies were also found in AMI patients. The expression frequencies of TCR Vδ7 and TCR Vδ6 in AMI patients were significantly lower than those in healthy controls. The high clonal expansion frequencies of the TCR Vδ8, Vδ4 and Vδ3 were determined in AMI patients. High expression of Foxp3 gene was found in AMI PBMCs, while high expression of IL-17A was found in AMI γδ+ cells.

**Conclusions:**

Restrictive expression of TCR γδ repertoire and alteration expression of IL-17A gene are the important characteristics of γδ T cells in AMI patients, which might be related to the immune response and clinical outcome. γδ T cells might play a key role in the pathological progress of AMI and associated with the IL-17A mediated pathway.

## Background

Although the early and effective primary percutaneous coronary intervention and thrombolytic therapy have greatly improved the survival and cardiac function in patients with acute myocardial infarction (AMI), AMI is still a major cause of morbidity and mortality throughout the world, accounting for 7 and 5% of the global burden of disease in males and females, respectively [1]. It is well known that the rupture or erosion of atherosclerotic plaques is the main cause of acute coronary syndrome (ACS). Atherosclerosis, once considered to be a mild accumulation of lipid in the arterial wall, is complex and poorly understood considering its lesion and development. In addition, JUPITER studies have showed that even those healthy participants without traditional risk factors, but with increased high-sensitive-C reactive protein (hsCRP), benefited from statin in therapy [2]. So it is thought that inflammatory reaction is involved in the process of atherosclerosis. Activation of inflammatory cells plays a key role in the pathogenesis of ACS [3]. Studies have showed that innate and acquired immune markers such as hsCRP were associated with the progression of atherosclerosis [4, 5], which indicated that both innate and adaptive immunity contributed to the development of the atherosclerosis and its complications [6]. And innate inflammatory mediators were also found to be up-regulated during and after AMI, suggesting that myocardial infarction (MI) mobilizes not only a sterile nonspecific inflammation, but also ‘adaptive’ immune responses to cardiac auto-antigens which are able to modulate the myocardial inflammation and fibrosis [5, 7].

T cells are the main components of cell mediated inflammation and have been demonstrated to be involved in the etiology and development of atherosclerotic plaques [8]. Activated T cells can release inflammatory mediators and procoagulants to improve the rupture and local thrombosis. Adaptive T-cell driven immune inflammatory response is involved in atherosclerosis and plaque instability, leading to ACS, including non ST-segment-elevation ACS and ST-segment-elevation acute myocardial infarction (STEMI) [9]. Nepoleao et al. found the decrease of CD3+ T lymphocyte count in STEMI patients at the day of STEMI onset, which was associated with plaque instability and disruption [10]. The function of activated T cells during the development of ACS can be downregulated by a special subgroup of T cells, the regulatory T cells (Tregs, a general term for a group of regulatory T cells which mediate immune suppression [11]). The latter suppress immune responses by inducing and maintaining immune tolerance [11].

Lymphocytes are divided into two main lineages in humans: B cells which generate antibodies for humoral immunity, and T cells that are responsible for cellular immune response. And T cells are further subdivided into two major populations characterized by the surface expression of a T cell receptor (TCR) α chain and a β chain (αβ T cells) or a γ chain and a δ chain (γδ T cells). Only 5–10% lymphocytes in peripheral blood are γδ T cells, which rise from 2 to 60% of CD3+ lymphocytes, a small subset of T cells in peripheral blood, and regulate the inflammation process in many diseases [12, 13]. In summary, γδ T cells are associated with the pathogenesis of coronary atherosclerotic heart disease (CAD), but the relationship between the development of AMI and γδ T cells is still not clear. In addition, the expression of γδ T cell subgroup populations in patients with AMI has not yet been reported. Based on previous literature and data, we hypothesized that γδ T cells play a key role in the pathogenesis and pathophysiology of AMI. The present study was designed to investigate the expression pattern and clonality of TCR repertoire of γδ T cells in AMI patients, analyze the expression levels of regulatory genes Forkhead box P3 (Foxp3) and Interleukin-17A (IL-17A), and characterize the correlation between γδ T cells and the pathogenesis of AMI.

## Methods

### Participants

25 patients (aged 64.4 ± 11.7 years, 17 males and 8 females) diagnosed with STEMI admitted to First Affiliated Hospital of Jinan University were enrolled. STEMI was defined by the presence of typically clinical symptoms (such as chest pain) associated with ST-segment elevation of > 2 mm in two contiguous chest leads or of ≥ 1 mm in two or more limb leads or left bundle branch block on a standard 18-lead electrocardiogram, and significantly elevated cardiac troponin-I (cTnI) above the recommended diagnostic threshold. All patients underwent a coronary angiogram at the admission and most of them with reperfusion by primary percutaneous intervention (PCI) concomitantly (only one patient underwent delayed PCI). All patients were clinically and biochemically characterized and were followed for a period of 29 months (22 ± 13 months) throughout patient clinic visits or telephone interview for recordings of clinical endpoints. The primary outcome was the composite of cardiovascular causes, recurrent nonfatal AMI, rehospitalization for heart failure, unstable angina pectoris or unscheduled PCI. Patients with a medical history or evidence of auto-immune disease, active inflammatory disease, malignancies, hematologic disorders, or current use of immunosuppressive agents including corticosteroids were excluded from the study. Fourteen healthy individuals (aged 57.3 ± 9.5 years, 8 males and 6 females) without clinical and electrocardiographic signs of CAD were included as controls. All of the procedures were conducted in accordance with the guidelines of the Medical Ethics Committees of the Health Bureau of Guangdong Province in China, and ethical approval was obtained from the Ethics Committee of the Medical School of Jinan University. Written informed consent was obtained from each participant.

### Sample collection

After obtaining participants’ consent, peripheral blood samples (PB) were obtained from AMI patients and healthy controls. And the blood samples of AMI patients were collected within 24 h since the onset of chest pain. Peripheral blood mononuclear cells (PBMCs) were isolated from peripheral blood (PB) samples by Ficoll-Hypaque gradient centrifugation. For the AMI patients, venous plasma sample and serum sample were also obtained for measurement of cardiac troponin I (cTnI) and creatinine kinases using an Abbott ARCHITECTi2000 Full Automatic Particle Chemiluminescence Immunoassay Analyzer, and of total cholesterol, triglycerides (TG), high-density lipoprotein cholesterol, and LDL-C, using an AU600 Biochemistry Analyzer (Olympus, Shizuoka, Japan).

### γδ T cell sorting

The γδ T cells in the PB from 25 AMI patients and 14 healthy controls were sorted using γδ monoclonal antibodies and the MACS magnetic cell sorting technique (Miltenyi Biotec, Bergisch Gladbach, Germany).

### Real-time quantitative polymerase chain reaction (RQ-PCR)

Total RNA of PBMCs and γδ T cells were extracted using TRIzol RNA extraction Kit according to the manufacturer’s protocol (ThermoFisher Scientific, CA, USA). High-Capacity cDNA Reverse Transcription Kit (ThermoFisher Scientific, CA, USA) was applied for reverse transcription so as to synthesize the first-strand cDNA. The quality of cDNA was analyzed by reverse transcription PCR (β_2_ microglubin (β_2_M) gene amplification). The primer sequences of the transcription factor Foxp3, IL-17A, and TCRVγ1–3 subfamily genes were listed in Table 1. RQ-PCR was performed in a volume of 20 μL containing 9 μL of 2.5× Real Master Mix, 0.5 μM of each primer and 1 μL of cDNA (Tiangen Biotech, Beijing, China). After the initial denaturation at 95 °C for 2 min, 45 cycles consisting of 95 °C for 15 s, 60 °C for 60 s and 82 °C for 1 s for plate reading were performed using MJ Research DNA Engine Opticon 2 PCR cycler (BIO-RAD, USA). The relative mRNA expression levels of relative genes in each sample were calculated according to the comparative cycle time (Ct) method [14].

**Table 1:** PCR primers of target genes and β_2_M gene

Primer	Sequence
Foxp3-for	5′-CTGACCAAGGCTTCATCTGTG-3′
Foxp3-back	5′-ACTCTGGGAATGTGCTGTTTC-3
TCR Vγ1-for	5′-TACCTACACCAGGAGGGGAAG-3′
TCR Vγ2-for	5′-GGCACTGTCAGAAAGGAATC-3′
TCR Vγ3-for	5′-TCGACGCAGCATGGGTAAGAC-3′
TCR Vγ-back	5′-GTTGCTCTTCTTTTCTTGCC-3′
IL-17A-for	5′-TCCCACGAAATCCAGGATGC-3′
IL-17A-back	5′-GGATGTTCAGGTTGACCATCAC-3′
β_2_M-for	5′-TACACTGAATTCACCCCCAC-3′
β_2_M-back	5′-CATCCAATCCAAATGCGGCA-3′

### Genescan analysis for clonality of TCRVγ and TCRVδ subfamilies

Three TCR Vγ1–3 subfamily gene senseprimers and a single TCR Cγ reverse primer or eight TCR Vδ sense primers and a single TCR Cδ primer were used in an unlabeled PCR for amplification of the TCR Vγ and TCR Vδ subfamilies, respectively. The sequences of primers were described in our previous study [15]. Aliquots of the cDNA (1 μL) were amplified in 20 μL reactions using one of the three Vγ primers and a Cγ primer or one of the eight Vδ primers and a Cδ primer. The final reaction mixture contained 0.5 μM of the sense and antisense primers, 1× PCR buffer, 0.1 mM dNTPs, 1.5 mM MgCl_2_, and 1.25 U Taq polymerase (Promega, CA, USA). The amplification was performed on a DNA thermal cycler (BioMetra, Germany). After 3 min-denaturation at 94 °C, 40 PCR cycles at 94 °C for 1 min, 60 °C for 1 min and 72 °C for 1 min were performed followed by a final 6 min-elongation at 72 °C.

Aliquots of the unlabeled PCR products (2 μL) were subjected to a cycle of runoff reaction using fluorophore-labeled Cγ-FAM or Cδ-FAM primer respectively. The labeled runoff PCR products (2 μL) were heat-denatured at 94 °C for 4 min with 9.5 μL formamide (Hi-DiFormamide, ThermoFisher Scientific, CA, USA) and 0.5 μL of Size Standards (GENESCANTM-500-LIZTM, ThermoFisher Scientific, CA, USA), and the samples were then loaded on 3100 POP-4TM gel (Performance Optimized Polymer-4, ThermoFisher Scientific, CA, USA) and resolved by electrophoresis in 3100 DNA sequencer (ThermoFisher Scientific, CA, USA) for size and fluorescence intensity determination using Genescan software [16].

### Statistical analyses

In the study, independents-samples *t* test was performed to compare the biochemical markers, and the Student’s *t* test, Kruskal–Wallis, or Mann–Whitney U test was performed to compare the means of gene expression levels between two cell populations. One-way ANOVA analysis was performed to compare the mRNA expression levels among cell populations. Pearson correlation or Spearman’s rank correlation analysis was used to estimate the correlations. Multivariate Cox-regression Analysis was performed, included the following variables: age, gender, absolute number of γδ T cells in PB, γδ T cell clonal expansion, levels of cTnI, creatinine kinase, total cholesterol, TG, HDL-C and LDL-C, expression levels of Foxp3, IL-17A, and TCR Vγ1–3 genes in γδ T cells, and clinical status of AMI patient. Statistical analysis was performed using SPSS version 19.0 statistic software package (SPSS, Inc., Chicago, IL, USA) and GraphPad Prism 5.0 (GraphPad Prism Software Inc., San Diego, CA, USA). *P* < 0.05 was considered as statistically significant.

## Results

### Clinical characteristics of patients

The clinical characteristics of the patients with AMI and healthy controls were showed in Table 2. The biochemical data and results of coronary angiography at admission were showed in Table 3. All the patients underwent follow-up of 22 ± 13 (0.5–36) months. During the follow-up, one patient died of ventricular septal perforation, one of the AMI complications; and another patient died of cerebral hemorrhage 2 months after AMI. Three patients underwent unscheduled PCI because of unstable angina pectoris, and two patients returned to hospital for heart failure during follow-up.

**Table 2:** Baseline demographic data of enrolled subjects

	AMI	Healthy controls	*P* value
Number	25	14	NA
Age (years) (median ± range)	64.4 ± 11.7	57.3 ± 9.5	0.060
Gender (male)	17 (68%)	8 (57%)	NA
Arterial hypertension	18 (72%)	0	NA
Diabetes mellitus	6 (24%)	0	NA
Prior myocardial infarction	0	0	NA
Dyslipoproteinaemia	11 (44%)	7 (50%)	NA
History of smoking	9 (36%)	0	NA
WBC (× 10^9^/L)	10.85 ± 3.45	6.97 ± 2.17	0.001
Total cholesterol (mmol/L)	5.09 ± 0.97	5.23 ± 0.85	0.672
LDL-C (mmol/L)	3.16 ± 0.80	3.11 ± 0.64	0.884
HDL-C (mmol/L)	1.12 ± 2.56	1.52 ± 0.32	0.002

*AMI* acute myocardial infarction, *WBC* white blood cells, *LDL*-*C* low-density lipoprotein cholesterol, *HDL*-*C* high-density lipoprotein cholesterol, *hs*-*CRP* high-sensitivity C-reactive protein

**Table 3:** Biochemical and clinical data of the AMI patients

	AMI
Number	25
hs-CRP (mg/L)	11.13 ± 11.65
Cardiac troponin I (pg/mL)	24.78 ± 15.06
NT-proBNP (ng/mL)	1266.73 ± 1685.79
LVEF (%)	51 ± 9
Infarct-related artery (no.)	
LAD	24
RCA	4
LCX	6
LM	1
Killip at admission	
Killip 1	7
Killip ≥ 2	18

*NT*-*proBNP* N-terminal pro B-type natriuretic peptide, *LVEF* left ventricular ejected fraction, *LAD* left anterior descending branch coronary artery, *LCX* left circumflex artery, *LM* left main coronary artery, *RCA* right coronary artery

### Expression pattern and clonality of TCR γδ T cells in AMI patients

**Fig. 1:**
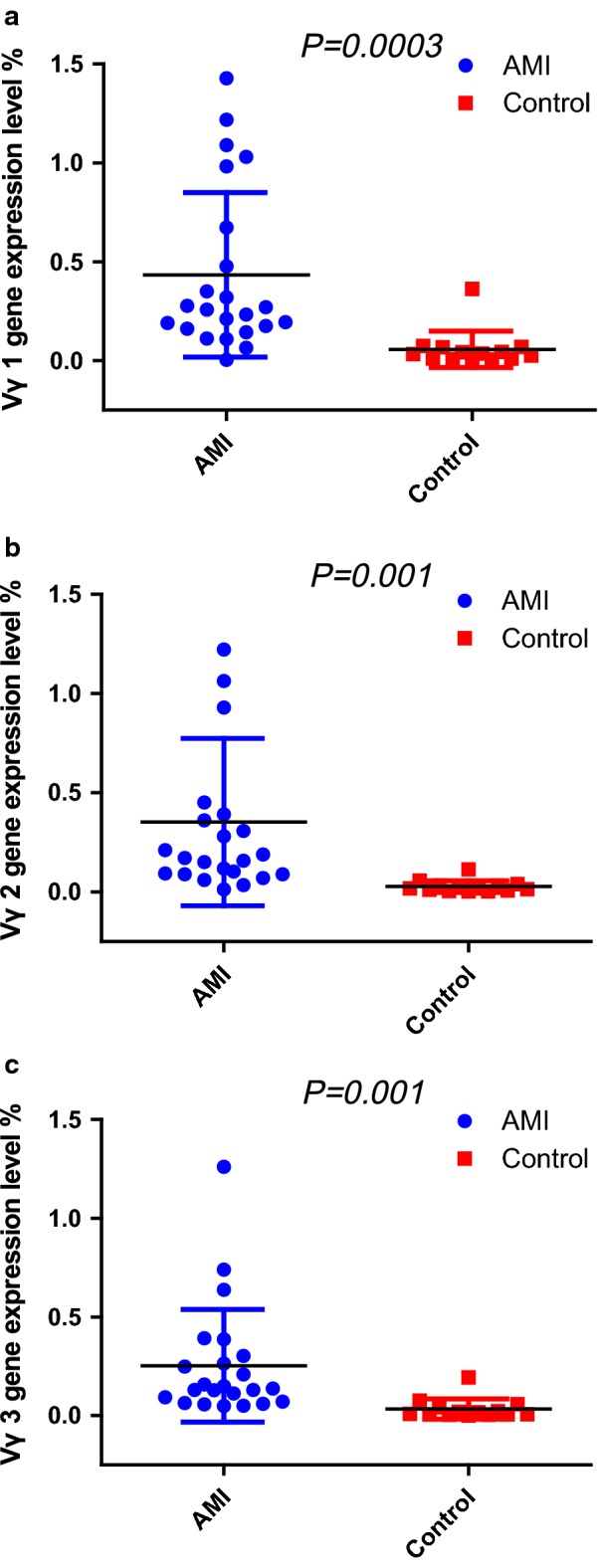
Quantitative analysis of mRNA expression levels of TCR Vγ subfamilies genes in γδ T cells of AMI patients and healthy individuals (Control). **a** Expression levels of TCR Vγ1 genes; **b** expression levels of TCR Vγ2 genes; **c** expression levels of TCR Vγ3 genes

**Fig. 2:**
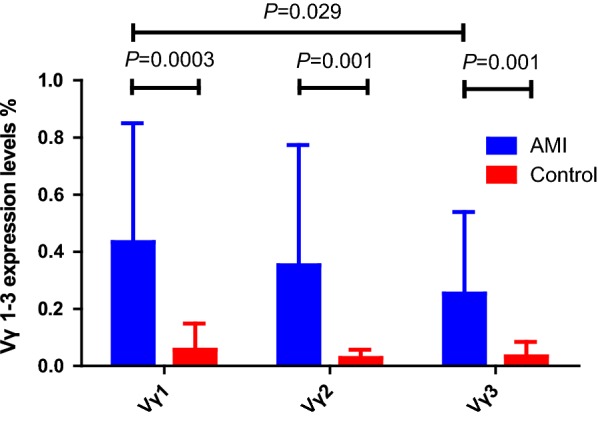
Expression pattern of TCR Vγ subfamilies (TCR Vγ1–3) genes in γδ T cells of AMI patients and healthy individuals (Control)

Quantitative analysis of mRNA expression levels of TCR Vγ subfamilies genes in γδ T cells of AMI patients and healthy individuals showed that the expression of TCR Vγ 1–3 genes were higher in AMI patients compared with that in healthy controls (0.43 ± 0.41% vs. 0.06 ± 0.09%, *P* = 0.0003 for Vγ1; 0.35 ± 0.42% vs. 0.03 ± 0.03%, *P* = 0.001 for Vγ2; 0.25 ± 0.29% vs. 0.03 ± 0.05%, *P* = 0.001 for Vγ3) (Fig. 1). The expression pattern was Vγ1 > Vγ2 > Vγ3 in patients with AMI, while Vγ1 > Vγ3 > Vγ2 in healthy controls (Fig. 2).

**Fig. 3:**
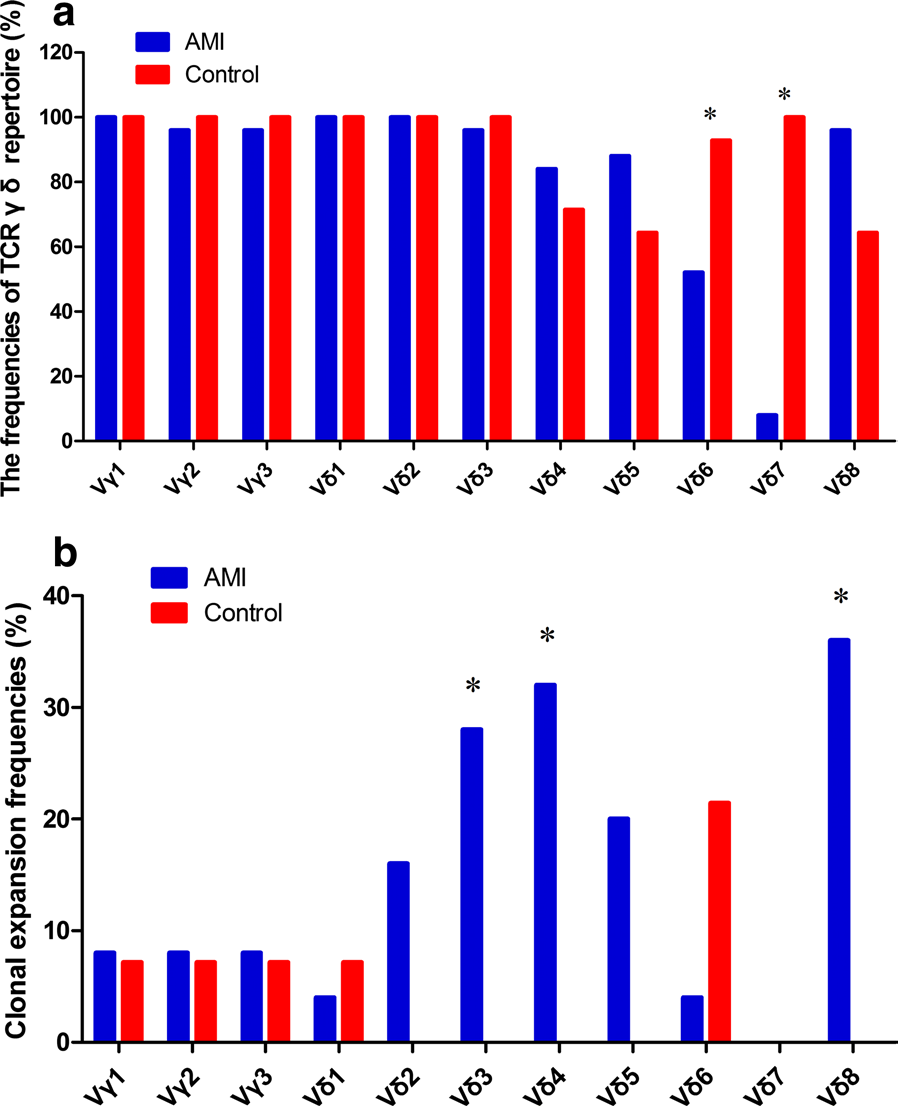
Expression clonality of TCR γδ T cells in AMI patients. **a** The frequencies of TCR γδ repertoire of AMI patients and healthy individuals (Control); **b** the clonal expansion frequencies of TCR Vγ and Vδ subfamilies of AMI patients and healthy individuals (Control). *P < 0.05

In this study, the CDR3 sizes of TCR Vδ (1–8) and Vγ (1–3) subfamily genes in sorted γδ T cells from AMI patients and healthy individuals were analyzed using RT-PCR and GeneScan. The mean value of the numbers of expressed TCR Vδ subfamilies in AMI patients (6.24 ± 0.72) was significantly lower than that in healthy individuals (6.86 ± 1.03, *P* = 0.034). The most frequently expressed subfamilies in the AMI patients were TCR Vδ1 (25/25, 100.00%), TCR Vδ2 (25/25, 100.00%), TCR Vγ1 (25/25, 100.00%), TCR Vδ8 (24/25, 96.00%), TCR Vγ2 (24/25, 96.00%), and TCR Vγ3 (24/25, 96.00%). And the frequencies of TCR Vδ7 (2/25, 8.00%) and TCR Vδ6 (13/25, 52.00%) were significantly lower than those in healthy individuals (14/14, 100.00%; 13/14, 92.86%) (*P* < 0.001 and 0.009, respectively) (Fig. 3a).

The majority of the TCR Vδ and Vγ subfamilies in the γδ T cells displayed polyclonal expansion with a Gaussian distribution of CDR3 lengths (multi-peaks) corresponding to apolyclonal rearrangement pattern. PCR product analysis produced a single dominant peak or double peaks, which demonstrated a skewed spectra type profile termed “oligoclonality” or “biclonality”, respectively. “Oligoclonality trending” is a classification with a profile between that of polyclonality and oligoclonality [17, 18]. The oligoclonally expanded γδ T cells were distributed in almost all of the TCR Vδ and Vγ subfamilies in the AMI patients with the exception of TCR Vδ7, and the most frequently oligoclonally expanded TCR Vδ and Vγ subfamilies were TCR Vδ8 (9/25, 36.00%), TCR Vδ4 (8/25, 32.00%) and TCR Vδ3 (7/25, 28.00%). The clonal expansion frequencies of TCR Vδ8, TCR Vδ4 and TCR Vδ3 subfamilies were significantly higher than those in healthy controls (*P* = 0.011, 0.018 and 0.029) (Fig. 3b).

### Expression of Foxp3 and IL-17A genes in γδ T cells

The mRNA expression levels of regulatory functional genes (Foxp3 and IL-17A genes) in γδ T cells of AMI patients and healthy controls were determined by RQ-PCR. It showed that the mRNA expression levels of IL-17A gene in γδ T cells of AMI patients (median: 0.0540%) were significantly higher than that of healthy controls (median: 0.0102%) (*P* = 0.0227). But no significant difference in the mRNA expression levels of Foxp3 gene was found between AMI γδ cells (median: 0.0101%) and normal γδ cells (median: 0.0054%) (*P* = 0.1185).

**Fig. 4:**
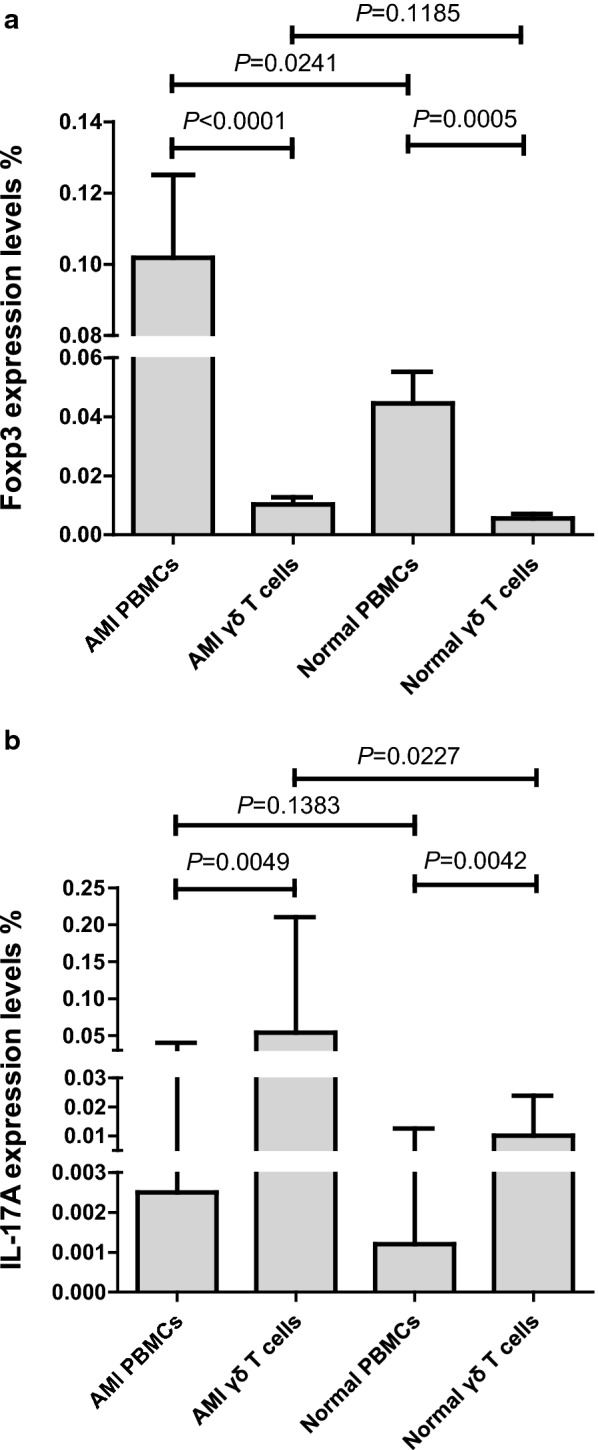
Subgroup population analysis of Foxp3 and IL-17A genes expression levels in AMI patients and healthy controls. **a** Comparison of Foxp3 gene expression levels among AMI γδ T cells, AMI PBMCs, normal γδ T cells, and normal PBMCs. **b** Comparison of IL-17A gene expression levels among AMI γδ T cells, AMI PBMCs, normal γδ T cells, and normal PBMCs

Subgroup population analysis was performed among AMI γδ cells, AMI PBMCs, normal γδ cells and normal PBMCs. Foxp3 was the highest expressed in AMI PBMCs (median: 0.0502%), but the lowest expressed in normal γδ cells (median: 0.0054%). Significant difference in the expression of Foxp3 was found between AMI γδ cells (median: 0.0101%) and AMI PBMCs (*P* < 0.0001), between AMI PBMCs and normal PBMCs (median: 0.0274%) (*P* = 0.0241), and between normal γδ cells and normal PBMCs (*P* = 0.0005) (Fig. 4a). IL-17A was the highest expressed in AMI γδ cells (median: 0.0540%), and the lowest expressed in normal PBMCs (median: 0.0013%). Significant difference in the expression of IL-17A was found between AMI γδ cells (median: 0.0540%) and AMI PBMCs (median: 0.0025%) (*P* = 0.0049), and normal γδ cells (median: 0.0102%) and normal PBMCs (median: 0.0012%) (*P* = 0.0042). No significant difference in the expression of IL-17A was found between AMI PBMCs and normal PBMCs (*P* = 0.1383) (Fig. 4b). Multivariate Cox-regression Analysis demonstrated that expression level of Foxp3 gene in AMI PBMCs was a common risk factor of the outcome of AMI [relative risks (RR) = 3.318, 95% CI 0.851–12.939].

No correlation was observed between the expression levels of Foxp3 and TCR Vγ1 (*P* = 0.363), between Foxp3 and TCR Vγ2 (*P* = 0.112), and between Foxp3 and TCR Vγ3 (*P* = 0.987). No correlation was also found between the expression levels of IL-17A and TCR Vγ2 (*P* = 0.078), and between IL-17A and TCR Vγ3 (*P* = 0.265). Interestingly, positive correlation was found between the expression levels of IL-17A and TCR Vγ1 (*P* = 0.047).

## Discussion

This is an exploratory study to investigate whether γδ T cells driven adaptive immunity is involved in the pathogenesis and pathophysiology of AMI. And we did find restricted expression of γδ T cell subsets and clonal expansion of some particular subsets in peripheral blood of patients with AMI. However, the relationship between abnormal expression of γδ T cell subsets and the clinical outcome of AMI is unknown.

TCR is the distinctive surface marker of T cells, and also an important molecule to recognize antigens, generate activated signals and mediate immune responses. TCR is composed of four peptide chains of α, β, γ and δ among which two different peptide chains make up heterodimer. According to the different peptide chains, the human peripheral blood T cells are divided into two subgroup populations-αβ T cells and γδ T cells. The αβ T cells account for most of the T cells in human peripheral blood, about 90–95% and are the main immune effector cells. The γδ T cells are only 1–10%, most of which distribute in the mucosa of the skin, the respiratory tract, the intestinal mucosa and the genitourinary system. The αβ T cells recognize peptides presented on the surface of antigen-presenting cells (APC) to CD8+ T cells by major-histocompatibility-complex (MHC) class I or to CD4+ T cells by MHC class II molecules. However, a sizeable subgroup population of CD3+ T cells carries γδ chains. The γδ T cells emerge as an “unconventional” subset of T cells in recent decades. It is now known as an evolutionarily conserved lymphocyte subset of T cells with diverse function, varying based on the species and disease state. In humans, γδ T cells could rise from 2 to 60% of total CD3+ lymphocytes based on immunological challenge [19]. Now it is known that γδ T cells support regeneration of epithelium as well as attract neutrophils just after tissue injury in order to remove necrotic epithelial cells in recent years, as they function not only in immunosurveillance but also in immunoregulation.

Human γδ T cells can be grouped into several discrete subsets based upon their different combinations of Vγ and Vδ chains at the variable (V) regions of the T cell receptors: Vγ1–3, Vδ1–8. We found all the expression of Vγ1, Vγ2, and Vγ3 genes were significantly increased in AMI patients compared with healthy controls (Fig. 2). And in our study, we did find the difference in expression pattern and clonality of TCR γδ T cells between AMI patients and healthy individuals. We found significantly restricted TCR γδ subfamilies expression in γδ T cells from AMI patients (the normal TCR γδ repertoire expression pattern is unrestricted). It is noteworthy that very low frequency of TCR Vδ7 subfamily was detected in AMI patients, but that was highly expressed in healthy individuals. The high clonal expansion frequencies of the TCR Vδ8, TCR Vδ4 and TCR Vδ3 were determined in AMI patients. We suggested that such expanded TCR Vδ8, TCR Vδ4 and TCR Vδ3 T cell clones might be reactive T cell clones directed against AMI. It might have certain clinical significance in the screening and prediction of AMI. However, this hypothesis requires confirmation with a larger cohort.

It is known that the immune response of the body mainly includes cellular and humoral immunity, and the specific cellular immunity mediated by T cells is particularly important. Decades ago, it was found that another important function of T cells is immune suppression or immunologic tolerance. Tregs, a general term for a cell population of regulatory T cells, can mediate immune suppression and normally express Foxp3 [20]. It is recognized as playing a critical role in maintaining immune system homeostasis and suppressing pro-inflammatory and “classic” immune response [7].

Atherosclerosis has been viewed as the bland accumulation of lipid in the arterial wall in recent decades, and evidences have indicated that myocardial infarction also launches a sterile unspecific inflammation over the past few years. But the researches of inflammation biology in cardiovascular disease mainly focus on protein mediators, such as cytokines and chemokines, or on small molecules, such as prostaglandins, reactive oxygen and nitrogen species. Little is known about the role of T cell adaptive immunity in the pathogenesis of AMI, although researchers have recognized the participation of different leukocyte classes in different stages of the process of AMI [21]. Data of animal experiments indicate that the number of naturally occurring CD4+CD25+ Tregs is associated with autoimmune diseases as well as atherosclerosis. Moreover, evidence showed that Foxp3 is the most specific marker for monitoring the development and function of CD4+CD25+ Tregs. Low expression level of CD4+CD25+Foxp3+ Tregs was thought to be involved in the development stages of human atherosclerosis [22]. Jia et al. reported that Tregs levels were decreased in ACS patients and associated with the severity of CAD as they assayed the demethylated Treg-specific demethylated region in Foxp3 in peripheral blood cells [8]. Furthermore, Mathes et al. demonstrated that CD4+Foxp3+ T-cells exerted damaged effects by enhancing myocardial ischemia–reperfusion injury in mice ischemia–reperfusion model without prior activation by MHC-II restricted autoantigen recognition [23]. In our study, it was demonstrated that Foxp3 gene was predominantly expressed in AMI PBMCs (higher than that in normal PBMCs and in AMI γδ cells). The expression levels of Foxp3 gene in AMI γδ cells were similar with that in normal γδ cells. As we known, Foxp3+ αβ T cells (CD4+CD25+Foxp3+ Tregs) have a higher expression ratio in PB compared with Foxp3+ γδ cells (the regulatory cell subsets of γδ T cells) in PB. So the dominant expression of Foxp3+ gene in AMI PBMCs was due to the high expression of Foxp3+ gene in CD4+CD25+Foxp3+ T cells of AMI patients. We also found the expression level of Foxp3 gene in AMI PBMCs was a common risk factor of the outcome of AMI (RR = 3.318). Foxp3 gene expression in PBMCs might play an important role in the progress of AMI patients. But further research needs to be done.

Szczepanik et al. reported that γδ T cells displayed dual directional regulatory function under different circumstance through secreting different cytokines [24]. Interleukin-17A (IL-17A, often referred to as IL-17) is proinflammatory cytokine which has received much immunological concern in the past few years as a key pathogenic factor in variously inflammatory diseases, including atherosclerosis and acute coronary syndrome, and is linked to autoimmune diseases [25–31]. It is mainly produced by CD4+Th17 cells according to this signature cytokine, but it can also be produced by other various cell types depending on health status and location, such as γδ T cells, natural killer cells, neutrophils, lymphoid tissue inducer cells [30]. It is now considered that IL-17 is expressed in human coronary and symptomatic carotid atherosclerotic lesions [32]. Jafarzadeh et al. reported that high serum level of IL-17 was associated with ischemic heart disease defined as AMI or unstable angina [25]. And Zhou et al. suggested that γδ T cells, instead of Th17 cells, were the primary source of IL-17 in infarcted heart, and the increase of IL-17 significantly expanded infarct size, worsened cardiac function, aggravated myocardial fibrosis and cardiomyocyte apoptosis in post-AMI patients. Conversely, genetic deficiency of IL-17 had the opposite effect. And they indicated that IL-17 induced cardiomyocyte apoptosis via the activation of p38 MAPK-p53-Bax signaling pathway [33]. Some animal models of atherosclerosis in vivo also showed similar results [34]. Nevertheless, Simon et al. suggested contrarily that elevation of IL-17 was associated with a better outcome in patients with AMI, indicating that IL-17 was a protective regulatory cytokine in CAD, and even an important modulator on vascular inflammation [28]. In this study, IL-17A expression was significantly increased in AMI patients, and especially higher in AMI γδ cells compared with AMI PBMCs and healthy controls with statistical difference. In addition, very low expression of IL-17A was found in PBMCs from healthy controls. And the highest expression of IL-17A gene in AMI γδ cells suggested that γδ cells producing IL-17 might associate with AMI. These results were consistent with previous literatures.

There are still many unexplained discoveries in this study that need to be further studied and discussed. As for the source of IL-17 in clinical AMI, although we found high expression of IL-17A in γδ T cells in AMI, which is consistent with a few literatures, further clinical studies in larger sample of AMI patients and animal experiment researches are needed. And how IL-17 functions in AMI? Proatherogenic or protective function? The perspectives of present literatures are contradictory. Liuzzo et al. suggested that IL-17 could be both proatherogenic and protective due to the microenvironment in which it is located and the producing of IL-17 and costimulating factors [35]. We indicated that IL-17 might have protective effects on AMI. But how abou t unstable angina pectoris and atherosclerosis? Subsequently, the mechanism of γδ T cells and IL-17 function needs to be further studied.

## Conclusion

In the study, we focused on the role of γδ T cells in the pathogenesis of AMI and found restrictive expression of some particular subfamily genes in AMI patients. Nevertheless, we also observed changes of expression level of regulatory functional genes, Foxp3 and IL-17A. These are the important characteristics of γδ T cells in AMI patients, which might be related to the immune response and clinical outcome. Even though the immunologic function of γδ T cells in AMI is unknown, an important role of γδ T cells can be speculated. Therefore, these findings may well reveal novel therapeutic options for those who suffered from AMI. Furthermore, we found that expression level of Foxp3 gene in AMI PBMCs was a common risk factor of the outcome of AMI, which need to be further confirmed by increasing sample size. Unlike CD4+CD25+Foxp3+ Tregs, which dominate hematological system tumors and autoimmune diseases, γδ T cells play a key role in the pathological progress of AMI and it may be associated with the IL-17A mediated pathway, but the specific mechanism is unknown. In summary, it suggested that Foxp3 in PBMCs and IL-17A in γδ T cells may be the early diagnostic factors and predictors of AMI.

## Abbreviations

ACS: acute coronary syndrome
AMI: acute myocardial infarction
CAD: coronary atherosclerotic heart disease
STEMI: ST-segment-elevation acute myocardial infarction
TCR: T cell receptor
RQ-PCR: real-time quantitative polymerase chain reaction
PBMCs: peripheral blood mononuclear cells
Foxp3: the transcription factor Forkhead box P3
IL-17(A): interleukin-17(A)
